# Investigation of the Importance of Climatic Factors in COVID-19 Worldwide Intensity

**DOI:** 10.3390/ijerph17217730

**Published:** 2020-10-22

**Authors:** Ploutarchos Tzampoglou, Dimitrios Loukidis

**Affiliations:** Department of Civil & Environmental Engineering, University of Cyprus, 1678 Nicosia, Cyprus

**Keywords:** COVID-19, weather, sociodemographic factors, response measures

## Abstract

The transmission of the severe acute respiratory syndrome coronavirus 2 (SARS-CoV-2) and the severity of the related disease (COVID-19) are influenced by a large number of factors. This study aimed to investigate the correlation of COVID-19 case and death rates with possible causal climatological and sociodemographic factors for the March to May 2020 (first wave) period in a worldwide scale by statistically processing data for over one hundred countries. The weather parameters considered herein were air temperature, relative humidity, cumulative precipitation, and cloud cover, while sociodemographic factors included population density, median age, and government measures in response to the pandemic. The results of this study indicate that there is a statistically significant correlation between average atmospheric temperature and the COVID-19 case and death rates, with chi-square test *p*-values in the 0.001–0.02 range. Regarding sociodemographic factors, there is an even stronger dependence of the case and death rates on the population median age (*p* = 0.0006–0.0012). Multivariate linear regression analysis using Lasso and the forward stepwise approach revealed that the median age ranks first in importance among the examined variables, followed by the temperature and the delays in taking first governmental measures or issuing stay-at-home orders.

## 1. Introduction

At the end of 2019, a new virus causing severe acute respiratory syndrome (SARS-CoV-2) was reported in Wuhan, China, and, due to its high human-to-human transmissibility [1], it quickly spread worldwide, resulting in the ongoing COVID-19 pandemic [2]. By the end of May 2020, which roughly signified the end of the first epidemic wave for most of the countries of the northern hemisphere, the total number of worldwide confirmed cases of COVID-19 was around six million, while the total worldwide deaths had reached 0.37 million [3], causing serious problems to the health systems of many countries.

**Figure 1:**
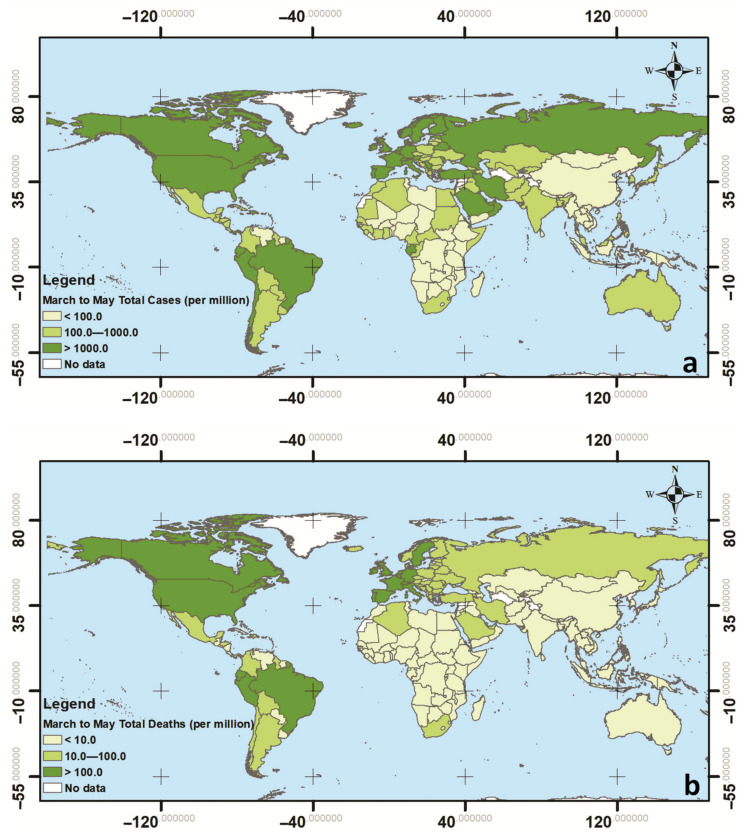
Worldwide distribution of (**a**) COVID-19 cases and (**b**) COVID-19-related deaths for the period of 1/3/2020–31/5/2020.

**Figure 2:**
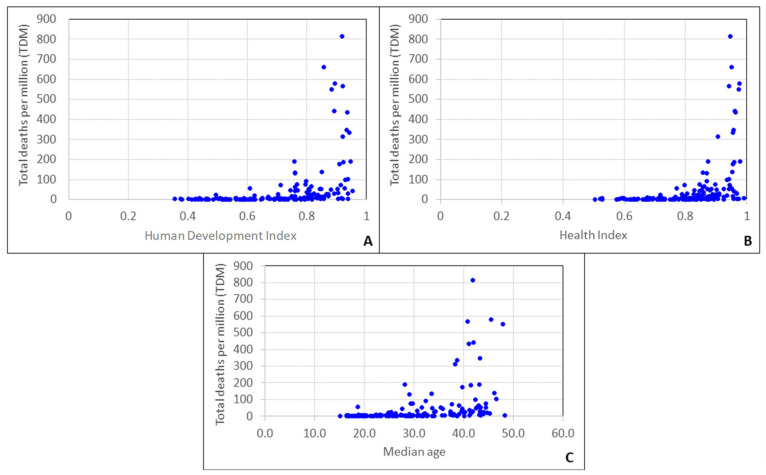
Variation of the total deaths per million for the period of 1/3/2020–31/5/2020 with (**A**) Human Development Index, (**B**) health index, and (**C**) median age (data for 167 countries).

By a simple examination of the pandemic data so far, one can easily notice the striking disparities (of orders of magnitude) in the reported confirmed case and death counts between countries (Figure 1). If certain socioeconomic indexes are considered alone, counterintuitive and irrational trends may be observed. For example, taking the Human Development Index (an index that includes the gross national income (GNI) per capita, the life expectancy, and the education level of each country) into account and comparing it with the total deaths per million of inhabitants (TDM) for the period of 3/2020–5/2020, it seems as if the countries of high development (and thus generally better health systems) are more susceptible to COVID-19 (Figure 2A). However, if we consider parameters directly associated with how aged is the population of a given country, e.g., the health index as defined by the United Nations Development Program (UNDP) or the median age (Figure 2B,C), it becomes obvious that the trend of Figure 2A can be explained, at least partly, by the fact that the populations of the more developed countries have a comparatively larger proportion of aged persons that are, in turn, more susceptible to develop heavy illness due to the SARS-CoV-2.

The vast ranges of case and death rates observed in Figure 1 could also be partially attributed to differences in the testing rates arising from the inherent difficulties faced when dealing with a new highly transmissible pathogen (e.g., access to adequate numbers of test kits and the availability of qualified laboratories). Nonetheless, large disparities in case and death rates are observed even among countries with similar economic prosperity, age structure and social characteristics. For example, in eurozone countries (the governments of which responded alike to the pandemic), the number of deaths per million of inhabitants in the 3/2020–5/2020 period ranged from 5 to over 800, i.e., two orders of magnitude difference. Thus, it becomes evident that the pathogen’s spread and impact are strongly affected by additional factors. These can be weather conditions, the promptness and mix of governmental response measures, air pollution, the volume of external travel, the frequency of use of public transportation, or other habitual and cultural/behavioral factors.

The effect of climatic factors on influenza epidemics has long being recognized [4,5,6,7,8,9]. More recently, after the onset of the new pandemic, a number of studies have been carried out to investigate the possible effect of weather on the spread of COVID-19. Some of these studies have focused on specific regions and countries, such as 122 cities in China [10], Wuhan, China [11], Mainland China [12], Jakarta, Indonesia [13], India [14,15], Iran [16], Brazil [17], Europe and United States [18], and Russia [19], while others have examined the effect of weather conditions on the pandemic at a global scale [20,21,22,23,24,25,26,27,28,29,30,31,32,33,34,35,36,37,38,39,40,41]. Smit et al. [42], who presented a comprehensive critical review of the relevant literature published in the first half of 2020, concluded that it is important for studies to include the effects of socio-economic factors and non-pharmaceutical interventions (such as government response measures) in their analysis.

This paper attempts to shed light on the degree of influence of climatic factors, namely temperature, relative humidity, precipitation, and cloud cover, on the intensity of the COVID-19 epidemic by examining the data of reported cases and deaths on a worldwide scale for the period of 3/2020–5/2020 (first wave) while simultaneously considering other important factors (social, economic, government response) that are expected to have a strong effect. First, one-to-one correlations are examined between the total numbers of deaths or cases (epidemic effects) and probable causal variables. Then, interference between causal variables is taken into account in order to reveal the actual degree of influence of the climatic factors in relation to sociodemographic ones and those related to government response. The results of this study, which help understand the relationship between the spatial distribution of the climatic conditions and the severity of COVID-19 on a worldwide scale, could be useful to decision-making authorities for the assessment of the pandemic risk and the management of the response measures.

## 2. Data Collection

The epidemiological data of total cases and total deaths due to COVID-19 in each country were taken from European Commission (EC) [43], Beltekian et al. [44], and Hale et al. [45] for the period between 1/3/2020 and 31/5/2020. These were used to calculate case and death rates as the total cases per million of inhabitants (TCM) and the total deaths per million of inhabitants (TDM) due to COVID-19 using the following formulas:

(1) $$\mathrm{TCM}=\frac{\mathrm{Total}\;\mathrm{number}\;\mathrm{of}\;\mathrm{cases}\;\mathrm{on}\;31/5/2020\;-\mathrm{Total}\;\mathrm{number}\;\mathrm{of}\;\mathrm{cases}\;\mathrm{on}\;1/3/2020}{\mathrm{Country}\;\mathrm{population}\times 10^{-6}}$$

(2) $$\mathrm{TDM}=\frac{\mathrm{Total}\;\mathrm{number}\;\mathrm{of}\;\mathrm{deaths}\;\mathrm{on}\;31/5/2020\;-\mathrm{Total}\;\mathrm{number}\;\mathrm{of}\;\mathrm{deaths}\;\mathrm{on}\;1/3/2020}{\mathrm{Country}\;\mathrm{population}\times 10^{-6}}$$

Furthermore, data about the median age and the government response measures taken in each country to reduce the spread of the pandemic—namely the overall stringency index of the government measures and the dates of officially recorded first cases, the imposition of first measures, and the imposition of ‘stay-at-home’ order—were gathered from Beltekian et al. [44] and Hale et al. [45], while data on the Human Development Index (HDI), population, etc., were collected from the Global Data Lab (GDL) database originating from the United Nation Development Program and referring to the year 2018 [46]. 

**Figure 3:**
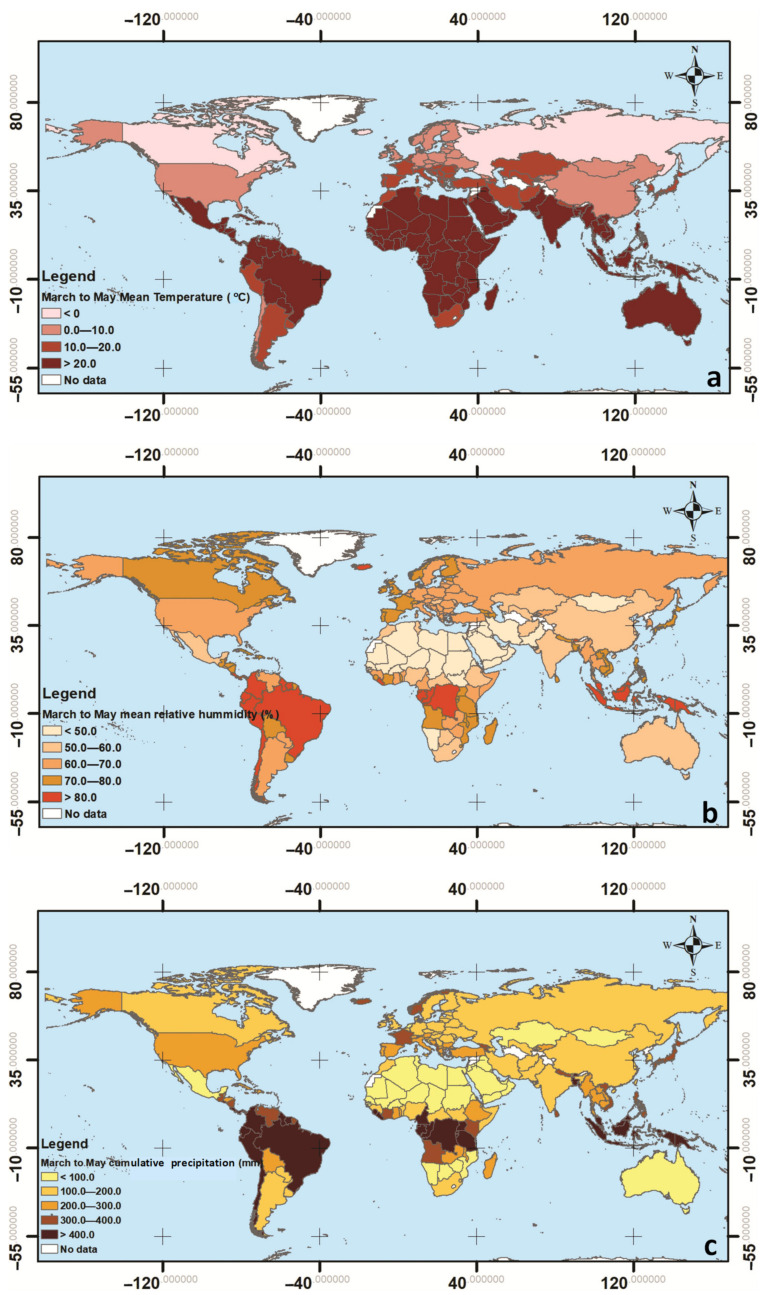
Worldwide distribution of (**a**) mean temperature, (**b**) mean relative humidity, and (**c**) cumulative precipitation for the period of 1/3/2020–31/5/2020.

Weather data, such as the monthly average atmospheric temperature (°C), monthly average relative humidity (%), and cumulative precipitation (mm), were collected from the Copernicus Program database [47] as raster files. These variables were estimated from climate reanalysis ERA-Interim and ERA5, depending on the source. The fraction of cloud cover, which is the proportion of a grid box covered by cloud (liquid or ice) and varies between 0 and 1, was taken from the Copernicus Program for a pressure level of 1000 hpa [48]. Then, the spatial analysis tool of the ArcGIS software (by ESRI) was employed to derive the spatial average of these variables across the entire territory of each country. After spatial averaging, the temporal average values were computed for the temperature, relative humidity, and cloud cover for the March 2020 to May 2020 period. Figure 3 presents maps showing the distribution of country average values of temperature, humidity, and cumulative precipitation.

## 3. Data Analysis

The collected data were divided in three groups of variables (Table 1). These were (i) the causal variables, i.e., the factors for which it was reasonable to assume that they may have an effect on the development of the pandemic; (ii) the effect variables, namely the total deaths and total cases per million of inhabitants; and (iii) the filtering variables, which were used to split and isolate datasets in order to minimize potential bias.

Regarding the filtering variables, due to many differentiations among countries arising from the difficulties faced during the early stage (first wave) of the pandemic that could affect the reported epidemiological data and, thus, the TCM and the TDM (e.g., the availability of test kits and equipment, the extent and intensity of testing campaigns, and consistency in reporting of cases and deaths), it was decided to not include in the analysis the least developed countries. For this purpose, the countries were sorted with respect to their Human Development Index (HDI), and those with an HDI < 0.7 were excluded from further statistical processing. The cut-off value of 0.7 coincides with the divider considered by the United Nations Development Program (UNDP) between the “high human development” and “medium human development” classes. This choice left the dataset with an adequately large number of countries (101 from the 169 for which data were available) and still contained the full spectrum of TDM values, including all countries with TDM > 100 (Figure 1a). Given the strong dependence of the TDM on the population age (Figure 1b,c), a second group was created by isolating the countries with a median age of 35 years and larger. The second group, which numbered 55 countries, turned out to be a subgroup of the first one, i.e., all countries in it also had an HDI > 0.7. It should be stressed that both groups covered the full range of climate types, from tropical to polar.

The causal variables included climatic and sociodemographic factors, as well as factors related to the government response to the pandemic. The climatic factors examined herein, namely the temperature, relative humidity, precipitation, and cloud cover, are generally thought to have a role in influenza epidemics, a connection investigated in previous studies mentioned in the introduction. It has been observed that cold weather aggravates the spread of respiratory infections [5,49,50,51], while clouds block solar UV light, which decreases the survivability of viruses in the outdoor environment [52,53]. It has long been debated whether absolute humidity or relative humidity should be considered as a proper driver of the influenza seasonality [6,54,55]. Marr et al. [55] pointed out that, nowadays, it is possible to explain the mechanism of how relative humidity may influence the transmission of the influenza virus (i.e., via its role in aerosol droplet size, evaporation, and salt concentration), but a plausible mechanism explaining how absolute humidity can directly affect droplet size has not yet been established.

Regarding social structural characteristics, given the fact that COVID-19 is associated with very high mortality among the elderly, we considered the median age and the percentage of the population that is above 65 years old to quantify how aged a society is. Moreover, high population density is also thought to be a risk factor [56]. It is reasonable to assume that delays in government response are affecting the outcome of the epidemic [57,58]. We considered two types of delays: the time difference between the officially recorded first case and (i) the imposition of a “stay-at-home” order and (ii) taking the “first measures.” According to Hale et al. [45], a stay-at-home order is defined as any of the following: (a) the recommendation of not leaving the house; (b) the requirement of not leaving the house except for work, daily exercise, grocery shopping, and ‘essential’ trips; (c) the requirement of not leaving the house but with the exceptions being much stricter (e.g., allowed to leave the house only once a week or only one person can leave at a time). The first measures varied in kind from country to country, although in most cases involved either public information/awareness campaigns, international travel restrictions, or the cancelling of public events. Finally, the Stringency Index is a composite measure that is based on nine response indicators, such as school closures, workplace closures, travel restrictions, and stay-at-home orders [44]. This index was averaged for a longer period (1/1/2020–31/5/2020) than the climatic factors because it was considered that measures taken even before the detection of first cases, such as banning travel from countries that exhibited active cases, reduced the progress of the pandemic during the 1/3/2020–31/5/2020 period.

### 3.1. Climatic Factors 

**Figure 4:**
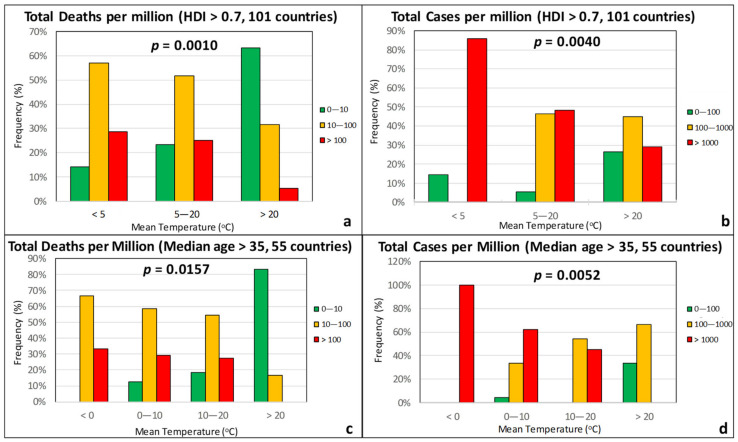
Frequency of total deaths per million (green: low, 0–10; orange: moderate, 10–100; red: high, >100) and total cases per million (green: low, 0–100; orange: moderate, 100–1000; red: high, >1000) as a function of the average temperature for countries with an HDI over 0.7 (**a**,**b**) and a median age over 35 years (**c**,**d**).

Figure 4 plots the frequency of TDM classes (low: <10; moderate: 10–100; high: >100) and TCM classes (low: <100; moderate: 100–1000; high: >1000) for three groups of average atmospheric temperature *T*. The ranges of values were selected in such a way that the number of countries in each group was similar. A strong dependence of both the TDM and the TCM on temperature is apparent, as the percentage of countries with high death rates (red group) decreases as *T* increases, while the percentage of countries with low death rates (green group) increases with *T* (Figure 4a,c). Similar trends are observed for TCM (Figure 4b,d). The vast majority of countries with *T* > 20 °C experienced the pandemic to a substantially lesser degree. The *p*-values from *χ*^2^ tests are also reported in the figures, with the null hypothesis being that the intensity of the epidemic (quantified by the TDM and the TCM) is independent from temperature. In all cases, the *p*-value is clearly less than 0.05 (the null hypothesis is rejected), suggesting a statistically significant correlation of the intensity of the epidemic with the temperature, especially in the case of the death rate. A small rise in *p*-values is noted when isolating the group of countries with a population median age of >35 years.

**Figure 5:**
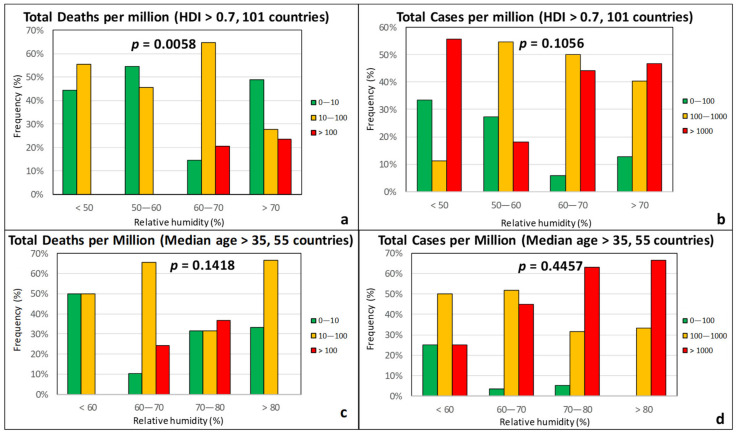
Frequency of total deaths per million (green: low, 0–10; orange: moderate, 10–100; red: high, >100) and total cases per million (green: low, 0–100; orange: moderate, 100–1000; red: high, >1000) as a function of the relative humidity for countries with an HDI over 0.7 (**a**,**b**) and a median age over 35 years (**c**,**d**).

**Figure 6:**
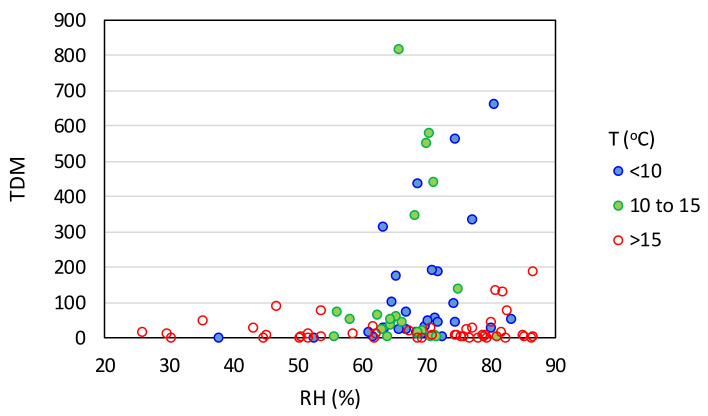
Total deaths per million as a function of the relative humidity and temperature (HDI > 0.7).

Based on Figure 5, it could be argued that there is a correlation of the TDM (and to a much lesser extent TCM) with the average relative humidity (*RH*). Nonetheless, the *χ*^2^ test *p*-values are higher in Figure 5 than in the case of temperature, suggesting a weaker correlation between relative humidity and intensity of the epidemic. From Figure 5a,c, it appears that the percentage of countries with a TDM > 100 (red group) is elevated when *RH* is larger than 60%. However, the group of countries with a TDM below 10 (green group) does not show a clear decreasing course with increasing *RH*, as their frequency is enhanced for *RH* > 70%. Similar observations can also be made in Figure 5b,d with respect to TCM. This can be partially attributed to the fact that many countries with an *RH* > 70% are located close to the equator or in the southern hemisphere, such as countries in Southeast Asia (e.g., Brunei, Indonesia, Malaysia, and Philippines) or around the Caribbean (e.g., Belize, Costa Rica, Cuba, and Jamaica), as well as New Zealand and Uruguay, and consequently had high temperatures in the examined time period. This observation suggests that *RH* does not influence the pandemic above a certain threshold of temperature. Most importantly, however, the epidemic severity appears to be strongly intensified if *RH* is in the 60–80% range provided that *T* is less than 15 °C, as can be better seen in Figure 6.

It is worth mentioning that studies on influenza [54,59,60] have shown that its transmissivity does not monotonically depend on *RH* if *T* lies between 10 and 25 °C, but it exhibits a local decrease (“valley”) inside the range of 40–60% (in which case the virus particles become unstable) and a local peak for *RH* between 60% and 75%. For higher values of *RH*, the transmissivity decreases sharply, regardless of the temperature, as aerosol droplets take on water and increase in size, thus settling more quickly due to gravity [59].

**Figure 7:**
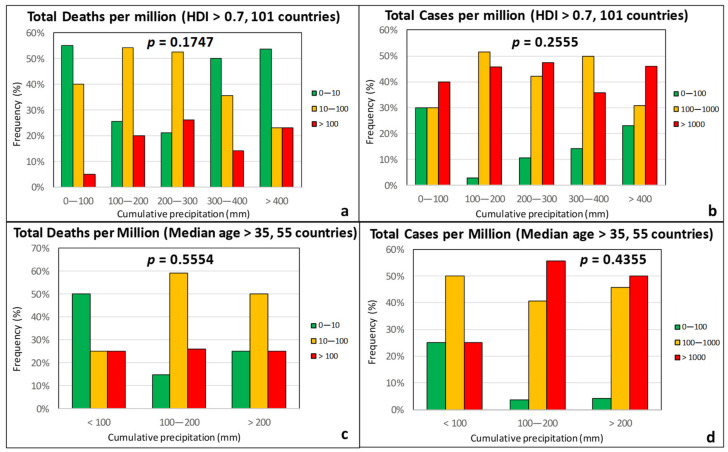
Frequency of total deaths per million (green: low, 0–10; orange: moderate, 10–100; red: high, >100) and total cases per million (green: low, 0–100; orange: moderate, 100–1000; red: high, >1000) as a function of the cumulative precipitation for countries with an HDI over 0.7 (**a**,**b**) and a median age over 35 years (**c**,**d**).

**Figure 8:**
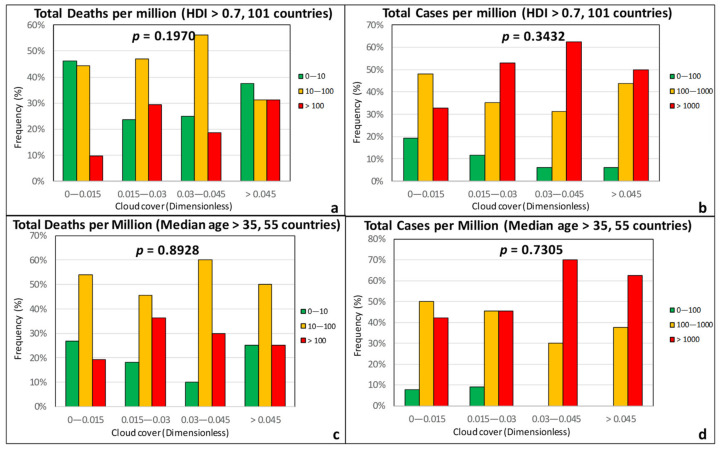
Frequency of total deaths per million (green: low, 0–10; orange: moderate, 10–100; red: high, >100) and total cases per million (green: low, 0–100; orange: moderate, 100–1000; red: high, >1000) as a function of the cloud cover for countries with an HDI over 0.7 (**a**,**b**) and a median age over 35 years (**c**,**d**).

Relative humidity, precipitation, and cloud coverage are climatic factors that are naturally related to each other. However, unlike *RH*, cumulative precipitation and cloud cover were found to correlate rather poorly with both the TDM and the TCM (Figure 7 and Figure 8). The *p*-values in all cases are high, indicating that there is no statistically significant correlation.

### 3.2. Sociodemographic Factors

**Figure 9:**
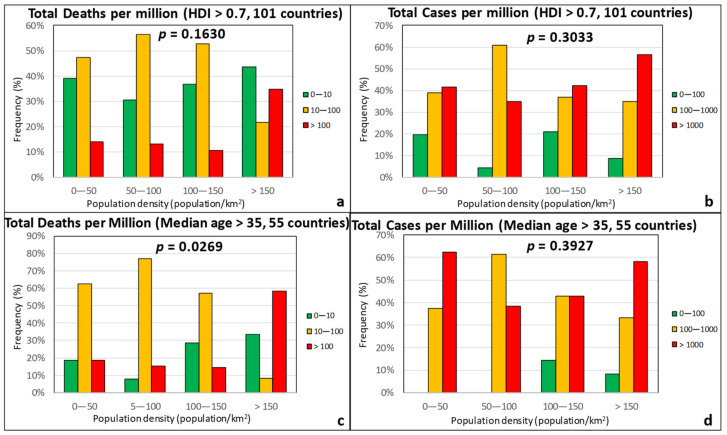
Frequency of total deaths per million (green: low, 0–10; orange: moderate, 10–100; red: high, >100) and total cases per million (green: low, 0–100; orange: moderate, 100–1000; red: high, >1000) as a function of the population density for countries with an HDI over 0.7 (**a**,**b**) and a median age over 35 years (**c**,**d**).

The contact numbers and transmission chains are larger in densely populated areas, and, thus, it would be reasonable to expect that countries with large population density would be more severely hit by the pandemic. Based on Figure 9, no specific trend can be discerned with respect to population density. This is also attested by the large *p*-values, which are in the 0.15–0.4 range. This admittedly counterintuitive observation is most likely caused by the treatment of data at a countrywide level, i.e., the population density treated herein was the total population of a country divided by its entire territorial area. For example, there are certain countries with a high TDM > 100 but with a population density below 50 inhabitants/km^2^ (e.g., Brazil, Canada, and Peru). Though these countries occupy vast areas, large parts of the population are concentrated in densely populated urban centers. A more detailed spatial analysis at first or second level administrative divisions may more clearly demonstrate and accentuate the role of population density. Nonetheless, the recent epidemiologic study for COVID-19 by Hamidi et al. [61], which was done at the level of US counties, also showed weak to no dependence of the epidemic severity on population density.

**Figure 10:**
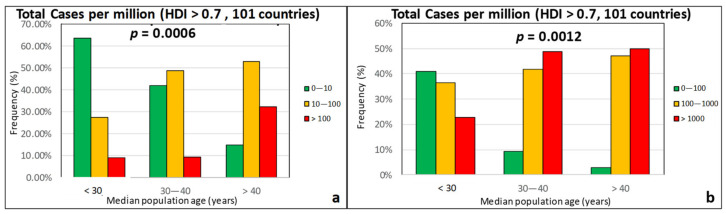
Frequency of (**a**) total deaths per million (green: low, 0–10; orange: moderate, 10–100; red: high, >100) and (**b**) total cases per million (green: low, 0–100; orange: moderate, 100–1000; red: high, >1000) as a function of the median age for countries with an HDI over 0.7.

**Figure 11:**
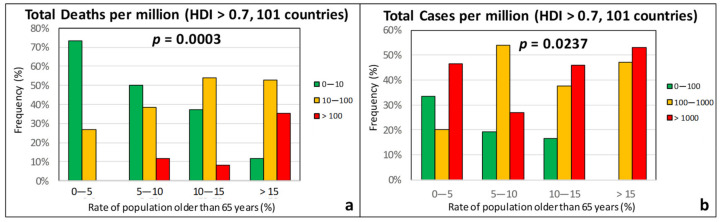
Frequency of (**a**) total deaths per million (green: low, 0–10; orange: moderate, 10–100; red: high, >100) and (**b**) total cases per million (green: low, 0–100; orange: moderate, 100–1000; red: high, >1000) as a function of the percentage of population with an age over 65 years for countries with an HDI over 0.7.

Figure 10 indicates that there is a strong correlation between both the TDM and the TCM with the median age of the population, with the *p*-values being well-below 0.05. This finding is well-expected, since the rate of COVID-19 cases is known to be comparatively low among young persons [62]. Given that the death rate, in particular, increases sharply with patient age, Figure 11 shows plots of the TDM and TCM frequencies as a function of the percentage of the population aged 65 years and older. In this case, the correlation with the TDM is even stronger than in Figure 10a, while the correlation with the TCM is weaker but still statistically significant.

**Figure 12:**
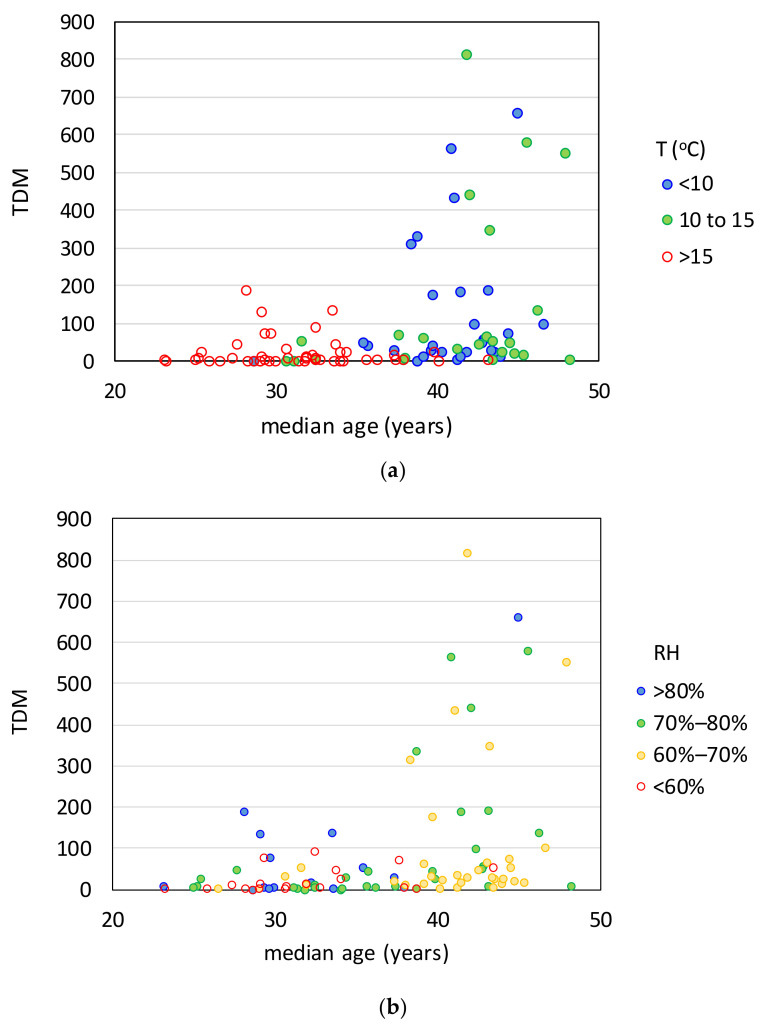
Total deaths per million as function of the population median age for different ranges of (**a**) temperature and (**b**) relative humidity (HDI > 0.7).

Thus far, the TDM and the TCM have shown strong correlations with the average temperature and median age, with similar *χ*^2^ test *p*-values. However, this does not necessarily mean that they are of similar significance. This is because of the correlation between these two causal variables. The first wave of COVID-19 unfolded during a period that was late winter and spring for the countries in the temperate zone of the northern hemisphere. Most of these countries happen also to be the most aged, while countries closer to the equator and most of the countries in the southern hemisphere have a relatively small median age. This fact is evident in Figure 12a, which plots the TDM as a function of the median age for three groups of countries separated based on the average temperature *T*. It can be seen that the majority of countries with *T* < 15 °C, which happen to be those most affected by COVID-19 (TDM > 100), have a median age larger than 35 years, while the opposite is true for the countries with T > 15 °C, most of which are located in South America, Africa, Middle East, and Southeast Asia. Similarly, most of the countries with average relative humidity in the 60–80% range have a median age larger than 35 years (Figure 12b). The interplay between the causal factors is addressed in Section 4, where their relative importance is examined using regression.

### 3.3. Government Response Measures

**Figure 13:**
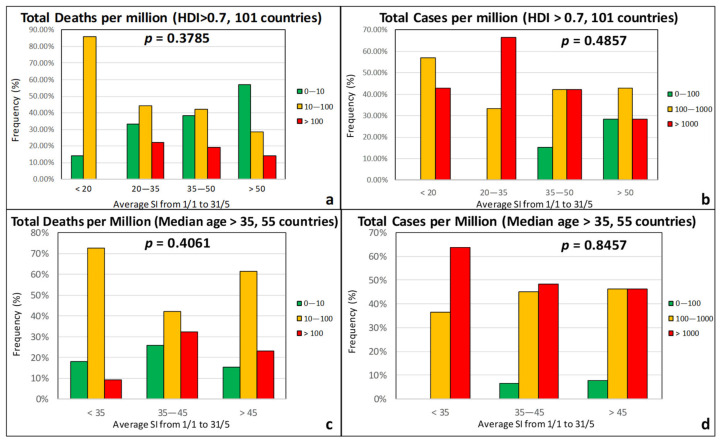
Frequency of total deaths per million (green: low, 0–10; orange: moderate, 10–100; red: high, >100) and total cases per million (green: low, 0–100; orange: moderate, 100–1000; red: high, >1000) as a function of the average Stringency Index for countries with an HDI over 0.7 (**a**,**b**) and a median age over 35 years (**c**,**d**).

**Figure 14:**
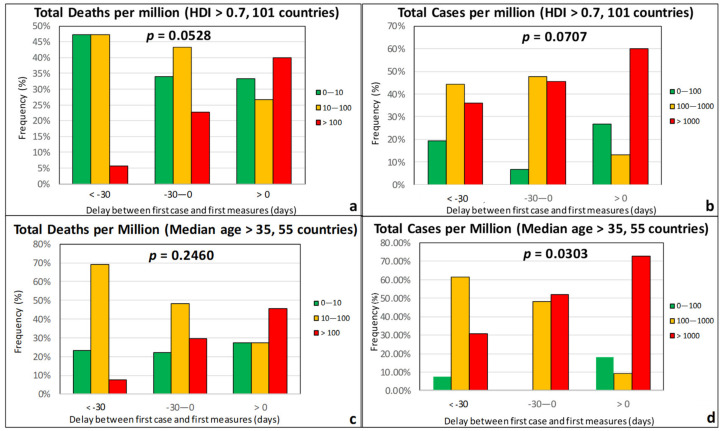
Frequency of total deaths per million (green: low, 0–10; orange: moderate, 10–100; red: high, >100) and total cases per million (green: low, 0–100; orange: moderate, 100–1000; red: high, >1000) as a function of the time difference between first case and first measures for countries with an HDI over 0.7 (**a**,**b**) and a median age over 35 years (**c**,**d**).

When considering the Stringency Index (SI) of the government response measures, as defined by Beltekian et al. [44], the *p*-values were found to be very high, indicating a weak correlation (Figure 13). On the contrary, the promptness of the governmental response seems to be instrumental in the containment of the epidemic. Based on Figure 14, countries that were proactive and responded by taking preventive measures one or two months before the officially recorded first case in the country (“negative” delay) experienced the first wave of COVID-19 less severely than countries that started taking the first measures after the detection of the first case.

**Figure 15:**
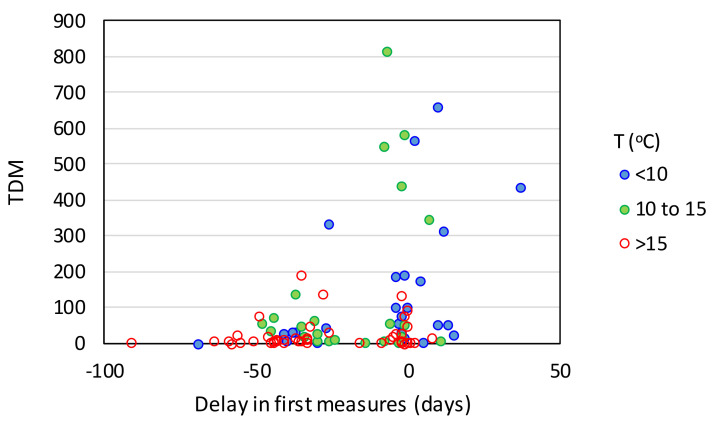
Total deaths per million as function of the time difference between the officially recorded first case and the first government measures for different temperature groups (HDI > 0.7).

When we isolated the countries with a median age >35 years (Figure 14b,c), the data suggested that the main role of the first governmental measures is to prevent a country from shifting from the group of moderate TDM and TCM values (orange) to the group with high values (red), while the frequency of countries that experienced the pandemic mildly is practically unaffected by the time difference between first measures and first case. Finally, according to Figure 15, the benefits of taking preventive measures early are rather independent from temperature, as a rising trend in the TDM with increasing time difference between the first measures and first case is observed in all temperature groups, especially those with *T* < 15 °C.

**Figure 16:**
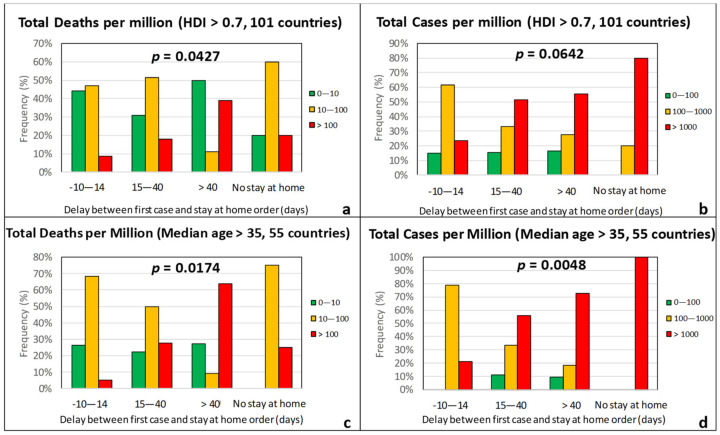
Frequency of total deaths per million (green: low, 0–10; orange: moderate, 10–100; red: high, >100) and total cases per million (green: low, 0–100; orange: moderate, 100–1000; red: high, >1000) as a function of the time difference between first case and the imposition of “stay-at-home” measure for countries with an HDI over 0.7 (**a**,**b**) and a median age over 35 years (**c**,**d**).

As seen in Figure 16, the frequency of countries that experienced the pandemic severely (TDM > 100 and TCM > 1000) increases steadily with increasing time difference between the official record of first case and the imposition of stay-at-home. With *χ*^2^ test *p*-values generally close or well-below 0.05, the delay in issuing stay-at-home order is judged as statistically significant in limiting the intensity of the epidemic. Moreover, with only very few exceptions, countries that did not impose stay-at-home fall in the groups of moderate (orange) and high (red) death and case rates. On the other hand, the frequency of countries with low death and case rates (green group) is practically unaffected by a delay in imposing stay-at-home, even of the order of two months. This may suggest that stay-at-home is mostly effective in preventing the escalation of the impact from moderate (orange) to high (red) in countries that are more susceptible to the epidemic due to other factors.

## 4. Multilinear Regression

### 4.1. Methodology

In the previous section, the epidemic effects were examined by considering each time a single causal variable, while the issue of possible interplay between certain causal variables was raised. In this section, we fit a linear equation combining all or groups of the causal variables, namely temperature, relative humidity, precipitation, cloud cover, population density, median age, government measures stringency index, delay in imposing first measures, and delay in imposing stay-at-home order. The percentage of population above 65 years old was not included in the set of variables since it exhibited a very strong correlation (*R*^2^ = 0.83) with the median age. The dependent variable was set to be the TDM given that it generally exhibited the strongest correlations with the causal variables, as shown in the previous section, and is less prone to bias caused by the testing numbers. Each causal variable *X_k_* enters the linear equation in the normalized form of (*X_k_* − *X_k_*_,min_)/(*X_k,_*_max_ − *X_k,_*_min_), where *X_k_*_,min_ and *X_k_*_,max_ are the minimum and maximum values of the variable in the dataset. With this normalization, all independent variables of the equation vary from 0 to 1. Hence, the linear equation to be fitted takes the following form:

(3) $$\begin{array}{l} \mathrm{TDM}=C_{0}+C_{1}\frac{T-T_{\min}}{T_{\max}-T_{\min}}+C_{2}\frac{RH-RH_{\min}}{RH_{\max}-RH_{\min}}+C_{3}\frac{PR-PR_{\min}}{PR_{\max}-PR_{\min}}+C_{4}\frac{CL-CL_{\min}}{CL_{\max}-CL_{\min}}+\\ +C_{5}\frac{PD-PD_{\min}}{PD_{\max}-PD_{\min}}+C_{6}\frac{MA-MA_{\min}}{MA_{\max}-MA_{\min}}+C_{7}\frac{SI-SI_{\min}}{SI_{\max}-SI_{\min}}+C_{8}\frac{FM-FM_{\min}}{FM_{\max}-FM_{\min}}+C_{9}\frac{SH-SH_{\min}}{SH_{\max}-SH_{\min}} \end{array}$$

where *T* is the average temperature (°C), *RH* is the average relative humidity (%), *PR* is the cumulative precipitation (mm), *CL* is the average cloud cover, *PD* is the population density (persons/km^2^), *MA* is the population median age, *SI* is the average stringency index, *FM* is the delay between first case and imposition of first measures (days), and *SH* is the delay between first case and stay-at-home order (days). To include the countries that did not impose stay-at-home in the regression analysis, their *SH* was calculated as the time difference between the officially recorded first case and the end of the investigated time period, i.e., 31/5/2020.

It must be stressed here that the purpose of the equation fitting was not to establish a predictive model but to discern the relative influence of the causal variables and rank them with respect to their importance. This is achieved herein using two conceptually different approaches: (i) the forward stepwise regression procedure and (ii) Lasso regression (along with its variation, the elastic net regression). The forward stepwise regression consists of performing consecutive multivariate linear regression calculations, starting with a single causal variable and adding in each step one more variable until the full set of *n* selected causal variables is exhausted [63,64]. With each step, the sum of squared errors (*SSE*) decreases. A measure of the importance of a variable *k* is the reduction increment in *SSE* induced by its inclusion in the equation (Δ*SSE_k_*). Because Δ*SSE_k_* depends on the order with which the variables are added to the linear model during the stepwise procedure, calculations are performed for all possible permutations of the causal variables (numbering *n*! in total) and the average Δ*SSE_k_* is calculated and subsequently used to rank the importance of each variable *k.* Moreover, it can be proven that, by normalizing Δ*SSE_k_* with the total sum of squares *SST*, one can obtain the increase in the coefficient of determination *R*^2^ caused by the inclusion of variable *k* in the equation (Δ*R*^2^*_k_*). To perform the calculations, the above procedure was scripted in MATLAB.

Lasso regression [65] introduces in the objective function a penalty term which is the sum of the absolute values of the linear equation coefficients *C_k_* (*k* = 1,2, …, *n*) times a user specified multiplier *λ* (i.e., *λ*Σ|*C_k_*|). The elastic net regression [66] is a generalized version of Lasso in which the penalty term is a weighted average of the sum of the absolute values of the coefficients and the sum of their squares (i.e., *λα*Σ|*C_k_*| + *λ*(1 − *α*)Σ*C_k_*^2^). If the weight factor *α* is set equal to 1.0, the regression is the same as the original Lasso, while as *α* decreases towards zero, the elastic net method tends to the ridge regression [67]. Herein, computations were performed for *α* = 1.0 and *α* = 0.9. By penalizing the presence of *C_k_*’s, these methods result in fittings that contain less non-zero coefficients (i.e., fewer causal variables than originally prescribed are effectively used) as *λ* is increased. Hence, a ranking of the importance of the causal variables could be obtained by repeating the regression calculations for a succession of increasing *λ* values. For very small *λ* values (negligible penalty), the fitted Equation (3) contains all nine causal variables. As *λ* increases, variables are successively dropped from the equation (their respective coefficients *C_k_* became zero). The larger the *λ* for which *C_k_* becomes zero (threshold value *λ_T_*), the greater is the importance of the respective variable. The Lasso and elastic net calculations were performed using the pertinent built-in function in MATLAB. 

### 4.2. Forward Stepwise Regression

The results of the stepwise linear regression approach are shown in Table 2 and Table 3 for a selection of combinations of causal variables. The average values of the increase in *R*^2^ due to the inclusion of each variable in the linear equation (Δ*R*^2^) from all permutations are given in Table 2, while Table 3 shows the corresponding regression coefficients. Combination A contained all nine variables, and the number of all possible permutations in this case was 362,880. Combinations B, C, and D excluded the cloud cover, the SI, and both of them at the same time, respectively, from the equation, because, as shown the previous section, they manifested the weakest correlation with TDM. Moreover, cloud cover is naturally correlated to a certain degree with humidity and precipitation. The cloud cover and SI were left outside the equation along with other variables for the remaining combinations presented in Table 2 and Table 3. Combinations E, G, I, and J contained only one of the three variables that are pertinent to atmospheric water (humidity, precipitation, and cloud cover). Finally, combinations H, I and J were set to contain only one variable pertaining to government response measures.

**Figure 17:**
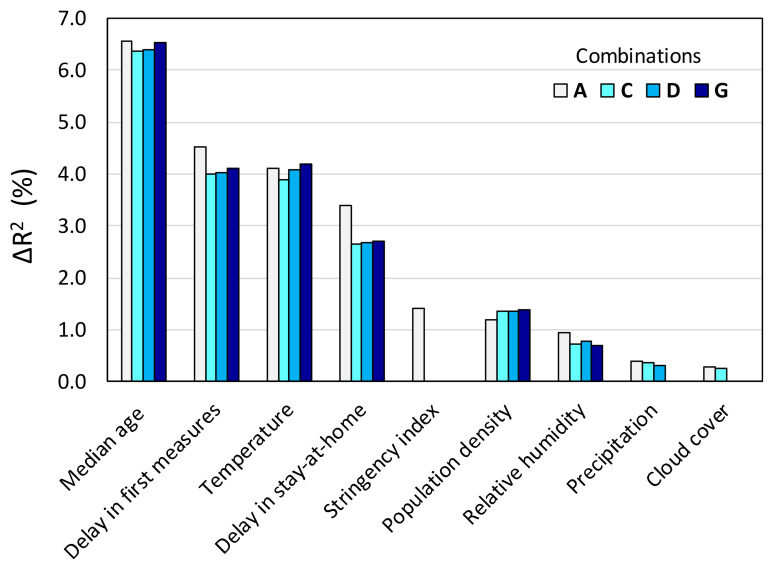
Rank of importance of causal variables based on stepwise regression.

In all combinations, the variable with the largest Δ*R*^2^ is clearly the median age (Table 2). The delay in imposition of first measures and the average temperature come second and third interchangeably depending on the combination, producing practically the same Δ*R*^2^ values, while the delay in stay-at-home ranks fourth (Figure 17). The variables related to atmospheric water appear to be of small to negligible importance, with the relative humidity being the most significant among the three. The very week correlation of TDM with cumulative precipitation and cloud cover is also manifested in the sign of the corresponding regression coefficients (*C*_3_ and *C*_4_), which, in certain combinations, turn out to be negative. It should be noted though that the actual significance of the relative humidity could be larger, given that it most likely has a non-monotonic effect on viral transmissibility, as discussed in Section 3.1, and that a linear regression inherently cannot capture non-monotonic dependencies. The same could be argued for the precipitation and cloud cover, given that these variables are interrelated with relative humidity.

As expected, the resulting coefficient for the temperature (*C*_1_) is negative in all combinations (Table 3), i.e., the TDM decreases with increasing average atmospheric temperature. The same would be anticipated for the coefficient for the SI (*C*_7_). However, *C*_7_ turns out to be consistently positive, implying that the TDM increases with the overall strictness of the government response. This counterintuitive result is probably because the SI is not purely a causal variable and can be actually considered an effect of the observed case and death rates. For example, if the epidemic in a given country escalates beyond control and overwhelms the health system, it is understandable that the government will ramp up the response and impose increasingly stricter measures in order to regain control.

The delay in issuing a stay-at-home order can also be argued to be a mixed cause/effect variable, i.e., that the delay is, to a certain extent, the outcome of how the epidemic is unfolding in a given country. For example, a state may choose not to impose stay-at-home measures at all if the case or death rates remain low due to other factors, or they may choose to impose stay-at-home measures late when certain thresholds are eventually exceeded. In case the delay in issuing a stay-at-home order was more an effect of the death rates rather than the cause, then it would correlate negatively with the TDM and the corresponding regression coefficient (*C*_9_) would be negative. As seen in Table 3, the resulting *C*_9_ values are always positive and the corresponding Δ*R*^2^ values are not negligible, suggesting that the stay-at-home measure is a factor instrumental in preventing escalation of the epidemic.

**Figure 18:**
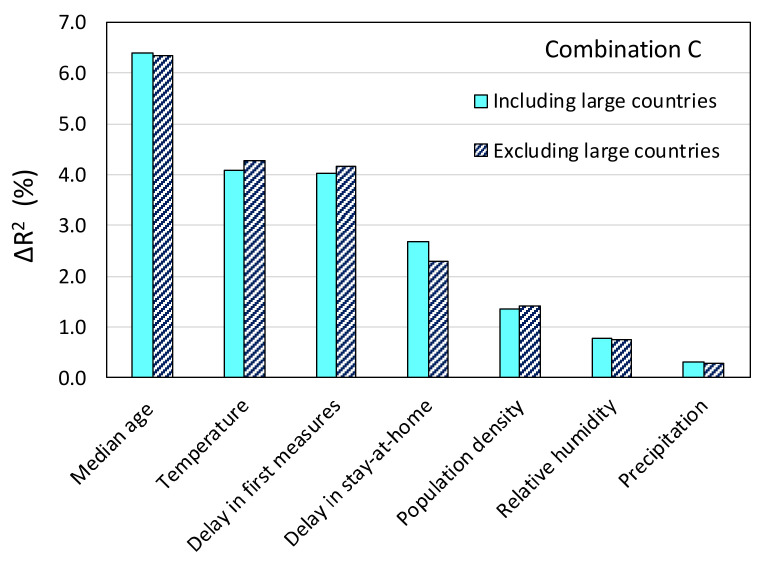
Effect of excluding large countries on the importance rank of causal variables resulting from stepwise regression.

This study relied on considering spatial averages across the entire territory of a country. It can be argued that such treatment may have been a source of bias in the analysis, especially in the case of large countries, as well as countries that span a wide latitude range in the temperate and arctic zones, in which case the differences in temperature (and possibly in other weather variables) are naturally large. Figure 18 shows the stepwise regression results for combination C when the six largest countries (Australia, Brazil, Canada, China, Russia, and USA) and two countries spanning a wide range of latitudes (Argentina and Chile) are removed from the dataset. It can be seen that exclusion of these countries has only a small effect on Δ*R*^2^, and the ranking remains the same as in the case of the complete dataset.

### 4.3. Lasso and Elastic Net Regression

**Figure 19:**
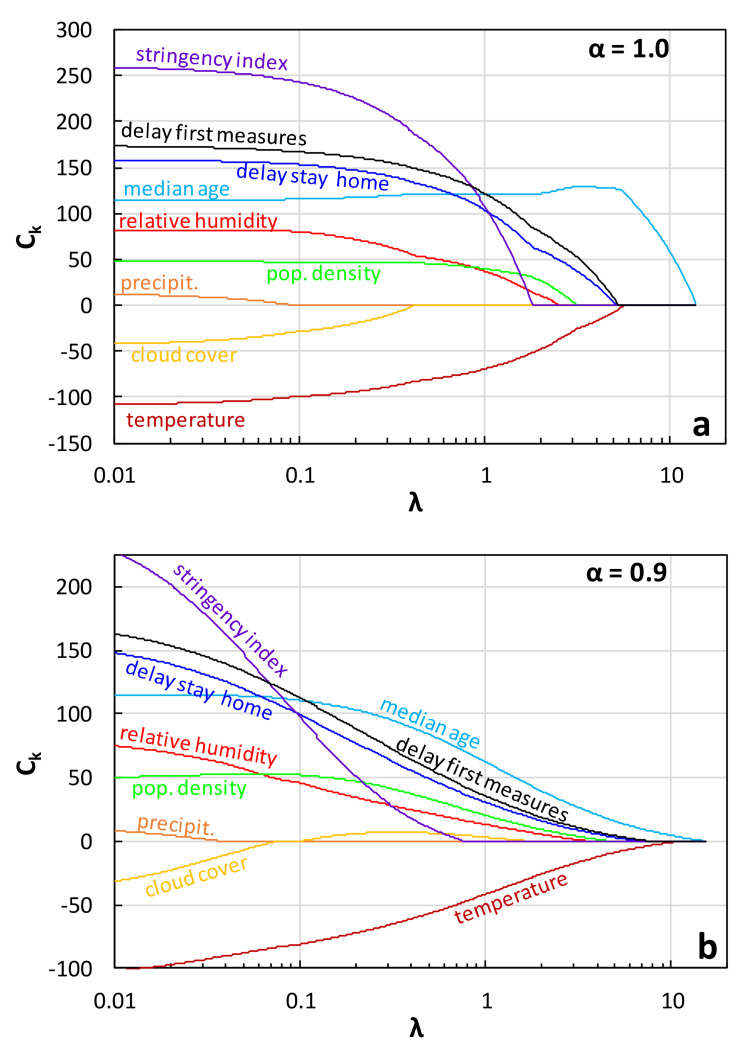
Trace plots of coefficients from: (**a**) Lasso regression and (**b**) elastic net regression.

**Figure 20:**
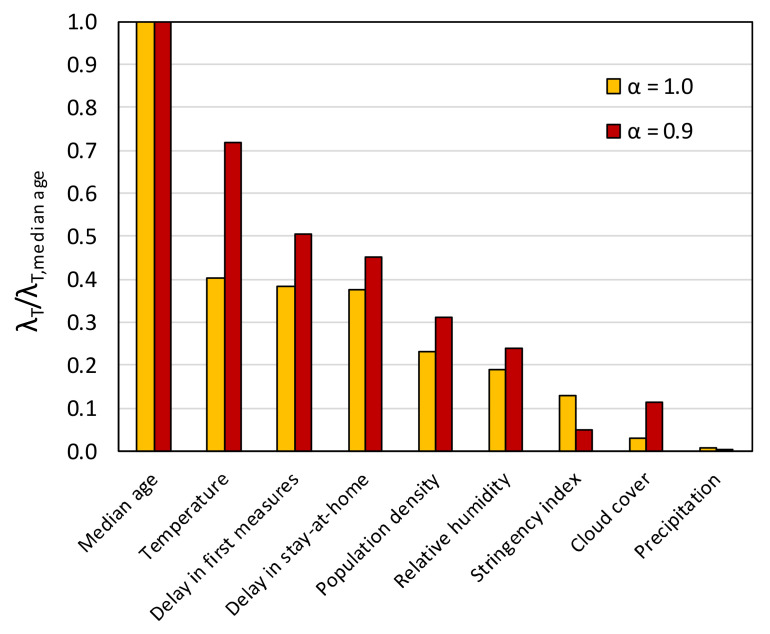
Rank of importance of causal variables based on Lasso and elastic net regression.

Figure 19 shows the variation of the coefficients *C_k_* with increasing *λ* (“trace plots”) from the application of Lasso regression (*α* = 1.0) and elastic net regression with *α* = 0.9, considering all nine causal variables of Equation (3). The *λ* values at which the curves reach the horizontal axis (*C_k_* = 0) are the threshold *λ_T_* values, which are plotted in Figure 20 (normalized with respect to the *λ_T_* for the median age). It can be observed that for *α* = 1.0 (Figure 19a), the curves dip more abruptly towards the horizontal axis and reach generally different *λ_T_* values than in the case of *α* = 0.9 (Figure 19b). Nonetheless, the inferred ranking of the importance of the variables is the same for both *α* values, with the exception of the stringency index and the cloud cover that switch places (Figure 20).

The rankings from the Lasso regression and elastic net regression generally agree with that from the forward stepwise regression. As in the case of the stepwise regression results (Table 2 and Figure 17), the median age turns out to be by far the most important variable, while the variables related to atmospheric water (RH, precipitation, and cloud cover) are clearly less significant compared to the government response measures (Figure 20). Furthermore, based on the Lasso results, the temperature, the delay in taking the first measures, and the delay in imposing stay-at-home order were found to be of almost equal importance, with the temperature being only slightly ahead. The differences among them are accentuated if *α* = 0.9. Figure 19 shows that the coefficient for the SI always remains positive (i.e., the severity of the epidemic increases with an increasing SI), an ambiguous outcome that, as discussed in the previous paragraph, probably stems from the fact that the stringency index is partially an effect variable. It should be stressed here that excluding the SI from Equation (3) did not change the ranking sequence seen in Figure 20.

**Figure 21:**
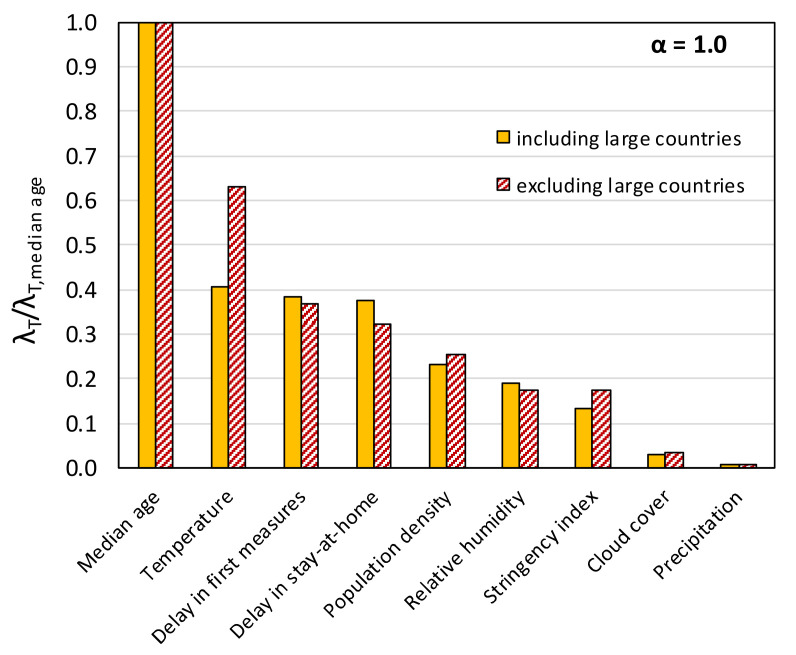
Effect of excluding large countries on the importance ranking of causal variables based on Lasso regression.

Finally, Lasso regression calculations were repeated excluding Argentina, Australia, Brazil, Canada, China, Chile, Russia, and USA in order to obtain results for a dataset in which the effect of spatial averaging across vast territories and wide range of latitudes was smaller. As seen in Figure 21, the exclusion of these countries caused insignificant changes in the *λ_T_*, with the exception of that corresponding to the temperature, the role of which appears elevated. Despite this, the ranking of the variables remains the same as in the case of the complete dataset.

## 5. Conclusions

Due to its high human-to-human transmissibility, COVID-19 quickly spread worldwide. However, discrepancies of orders of magnitudes have been observed among the countries in terms of case and death rates. To discern the role which climatic factors play in the local epidemic intensity in conjunction with sociodemographic factors and government response measures, atmospheric data were processed in Geographic Information System (GIS) and statistically analyzed for the period of the first wave of the pandemic.

Based on the results of the present study, among the climatic variables, the average temperature showed a strong correlation with both death and case rates. Its influence appears to be of at least equal importance to the promptness of the governmental response. Relative humidity was found to have a noticeable influence that was not easily discerned, probably due to its non-monotonic character. Nonetheless, the age structure of the population clearly emerged as the most important risk factor. On the contrary, our analysis found that population density correlated rather weakly with the epidemic intensity.

With respect governments’ response to COVID-19, the time difference between the first case detection in a given country and the imposition of first measures (which mainly consist of public information/awareness campaigns, international travel restrictions, and the cancelling of public events) appears to be instrumental in keeping case and death rates at moderate levels (less than 100 deaths and 1000 cases per million inhabitants). The present analysis suggests that the time difference between the occurrence of the first case and the issue of stay-at-home order also has a significant effect in preventing the escalation from moderate to high case and death rates but to a lesser degree compared to the promptness of first measures. 

As final remark, it must be kept in mind that in this study, the data were treated by taking countrywide spatial averages, which had a data-smoothing effect. Future investigations relying on a more detailed, regional discretization may more clearly elucidate the role of weather on COVID-19. The temporal averaging during the span of three months may also have obscured the actual degree of the dependence of the epidemic intensity on the climatic factors. For example, the initial cold period of March 2020 (colder than the average for the entire March–May period) in countries located in the temperate zone of the northern hemisphere may have seeded large numbers of early infections that then persisted as temperatures rose in April and May to cause lower reproduction numbers *R*. In this case, high COVID-19 case and death rates would be paired with artificially (due to averaging) larger temperature values. Such skewing is expected to be almost absent in the case of countries in which the weather variation during this three-month period was small, such as those that are located closer to the equator.

## Figures and Tables

**Table 1:** Variables considered in the statistical analysis.

Effect Variables	Causal Variables	Filtering Variables
Total Deaths per Million (TDM) Total Cases per Million (TCM)	*Climatic* Temperature (°C) Relative humidity (%) Cumulative precipitation (mm) Cloud cover *Sociodemographic* Population density (persons/km^2^) Median age (years) Percentage of Population over 65 (%) *Government response* Stringency IndexDelay between first case and first measures (days) Delay between first case and “stay at home” order (days)	Human Development Index (HDI) Median Age (years)

**Table 2:** Increase in coefficient of determination (Δ*R*^2^) due to inclusion of variables in Equation (3).

Variable	Combination									
	A	B	C	D	E	F	G	H	I	J
**Temperature**	0.041	0.042	0.039	0.041	0.042	0.037	0.042	0.045	0.046	0.047
**Relative humidity**	0.009	0.010	0.007	0.008		0.010	0.007	0.008	0.008	0.007
**Precipitation**	0.004	0.004	0.004	0.003	0.002	0.004		0.003		
**Cloud cover**	0.003		0.003							
**Population density**	0.012	0.012	0.014	0.013	0.015		0.014	0.013	0.013	0.016
**Median age**	0.066	0.067	0.064	0.064	0.067	0.069	0.065	0.067	0.068	0.076
**Stringency index**	0.014	0.014								
**Delay in first measures**	0.045	0.045	0.040	0.040	0.041	0.042	0.041	0.047	0.048	
**Delay in stay-at-home**	0.034	0.034	0.027	0.027	0.027	0.026	0.027			0.034

**Table 3:** Calculated values of regression coefficients.

Variable	Coefficient	Combination									
		A	B	C	D	E	F	G	H	I	J
**Temperature**	C1	−108.9	−88.9	−86.7	−89.7	−93.8	−60.6	−86.8	−103.9	−99.1	−90.9
**Relative humidity**	C2	82.2	79.1	34.8	34.9		62.7	46.1	34.7	54.2	40.6
**Precipitation**	C3	13.4	−17.9	11.3	16.3	45.0	−14.8		28.1		
**Cloud cover**	C4	−43.5		6.6							
**Population density**	C5	48.8	47.5	72.9	73.3	80.3		70.3	70.3	64.9	78.9
**Median age**	C6	113.8	120.2	109.2	108.1	112.6	125.7	106.4	110.1	107.1	127.7
**Stringency index**	C7	261.0	246.9								
**Delay in first measures**	C8	174.2	167.7	128.5	129.2	130.9	133.9	127.5	156.0	153.6	
**Delay in stay-at-home**	C9	158.8	157.3	94.7	94.4	94.3	91.4	95.5			122.1
**Intercept**	C0	−267.7	−270.4	−52.7	−50.4	−35.3	−78.2	−54.1	−31.5	−37.5	−3.9
